# Is use of a smuggler to cross the US-Mexico border associated with mental health problems among undocumented immigrants from Mexico?

**DOI:** 10.1371/journal.pgph.0002232

**Published:** 2023-08-14

**Authors:** Fernando A. Wilson, Jim P. Stimpson, Alexander N. Ortega

**Affiliations:** 1 Matheson Center for Health Care Studies, University of Utah, Salt Lake City, UT, United States of America; 2 Dornsife School of Public Health, Drexel University, Philadelphia, PA, United States of America; Northeastern University, UNITED STATES

## Abstract

Although numerous studies have found that Latine immigrants to the United States (US) have better health outcomes on average than persons born in the US, studies of persons living in Mexico have found that undocumented immigrants have worse health, especially those that were deported, compared to Mexican citizens that never migrated or migrated with authorization. However, the health outcomes of Mexican migrants using a smuggler to cross the US-Mexico border is a gap in the literature. We hypothesized that undocumented immigrant adults who used a smuggler to cross the US-Mexico border would be more likely to report mental health problems upon return to Mexico compared with undocumented immigrant adults that did not use a smuggler. We analyzed nationally representative, cross-sectional survey data of 1,563 undocumented immigrants currently living in Mexico. Most undocumented immigrants in the sample (87%) used a smuggler. Use of a smuggler by undocumented immigrant adults was associated with a 4.7% higher prevalence of emotional or psychiatric problems compared to undocumented immigrant adults that did not use a smuggler. We conclude that modality of ingress into the US is a risk factor for poorer mental health among undocumented immigrant adults.

## Introduction

Despite social and economic challenges, immigrants to the United States (US), have better health outcomes on average than persons born in the US [1–3]. However, the health advantage erodes over time. For example, allostatic load, a physiological index of chronic stress, has been found to increase as the number of years living in the US increases for Latine immigrants [4]. Much of what is known about immigrant health has focused on nativity, citizenship, and length of residence based on available data in state and national databases. The best source of data about undocumented immigrants in the US comes from the California Health Interview Survey, which has provided valuable insights into the health outcomes of Latine immigrants living in California [5, 6]. National estimates of the impact of social and policy barriers to public benefits, especially healthcare, on immigrant health have used imputation methods [7–9].

A long-term, nationally representative study of households in Mexico has provided important insights into the path to entering the US and the resulting socioeconomic and health outcomes [10–12]. One path for immigrants to become undocumented is to overstay their authorized temporary residency period under a valid US visa. To attain a legal visa is an arduous and expensive process for working-aged Mexican citizens, requiring proof of financial self-sufficiency or substantial assets. Immigrants who do not apply for or are turned down for a US visa and, consequently enter the US without authorization might use a smuggler, also known as a coyote, to cross the border, especially immigrants that have high socioeconomic vulnerability within Mexico [13–15]. Prior studies using the data from persons living in Mexico have found that undocumented immigrants have worse health, especially those that were deported, compared to Mexican citizens that never migrated or migrated with authorization [10–12]. However, the health consequences of using a smuggler represent an evidence gap.

To address this gap in knowledge, we analyzed a unique, publicly available, nationally representative survey of former US immigrants residing within Mexico and used a quasi-experimental method to compare the mental health of undocumented immigrants from Mexico who either used a smuggler or not to cross the US-Mexico border. We hypothesized that undocumented Mexican immigrants that utilized a smuggler to cross the border would be more likely to report mental health problems upon return to Mexico compared with undocumented Mexican immigrants that did not use a smuggler.

## Methods

The data for this study came from the Mexican Migration Project (MMP), which is an annual, nationally representative, cross-sectional survey of Mexican heads of households [16]. The survey has been in the field for four decades with approximately a 90% participation rate each year [16]. We pooled survey years from 2007–2019. Documentation of immigration status used included type of US visa, proof of US citizenship, or none or false documents if undocumented. During the study period, 1,563 undocumented immigrant adults (18 years of age and older) residing in Mexico were included in the analytical sample.

We downloaded this publicly available, de-identified data for free from the project’s website [16]. The authors of this paper were not involved in the collection of survey data and do not have an affiliation with the Mexican Migration Project. The Drexel University institutional review board determined that the study was exempt.

Our outcome measure was respondent reports of whether they ever had or currently have emotional or psychiatric problems, dichotomized as a yes or no response. The exposure variable was defined by whether the immigrant used a smuggler or not to cross the US-Mexico border. Covariates included survey year, age (18–34, 35–49, 50–64, 65 and older), sex (female versus male), marital status (married versus non-married), years of education (<6 years, 6 years, 7–11 years, 12 or more years), smoking status (ever smoked versus never smoked), obesity status (obese/overweight versus normal/underweight), home ownership, vehicle ownership, current ownership of a business, and number of US migrations (1 versus 2 or more).

**Fig 1:**
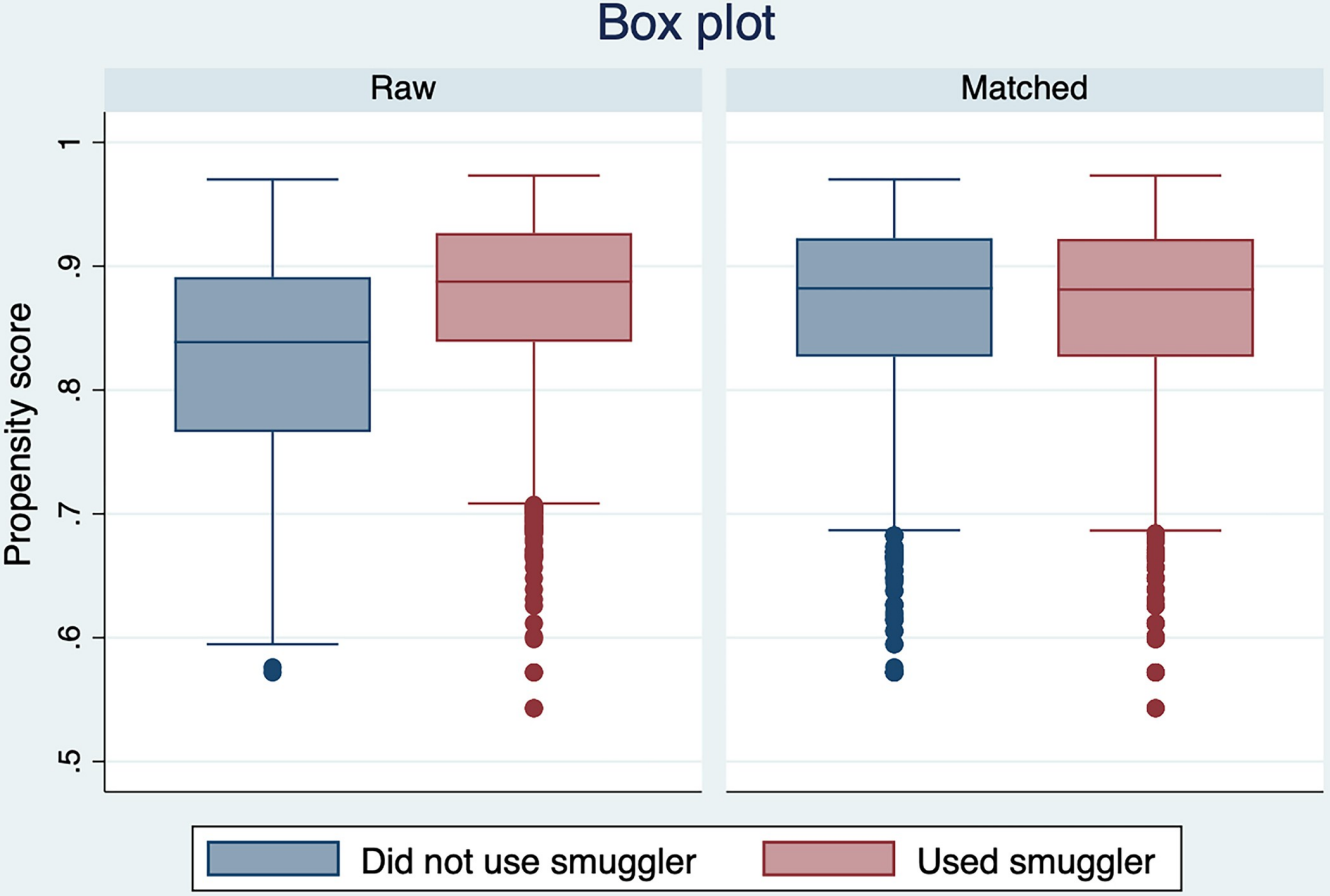
Balance box plot by use of a smuggler to cross the US-Mexico border.

**Fig 2:**
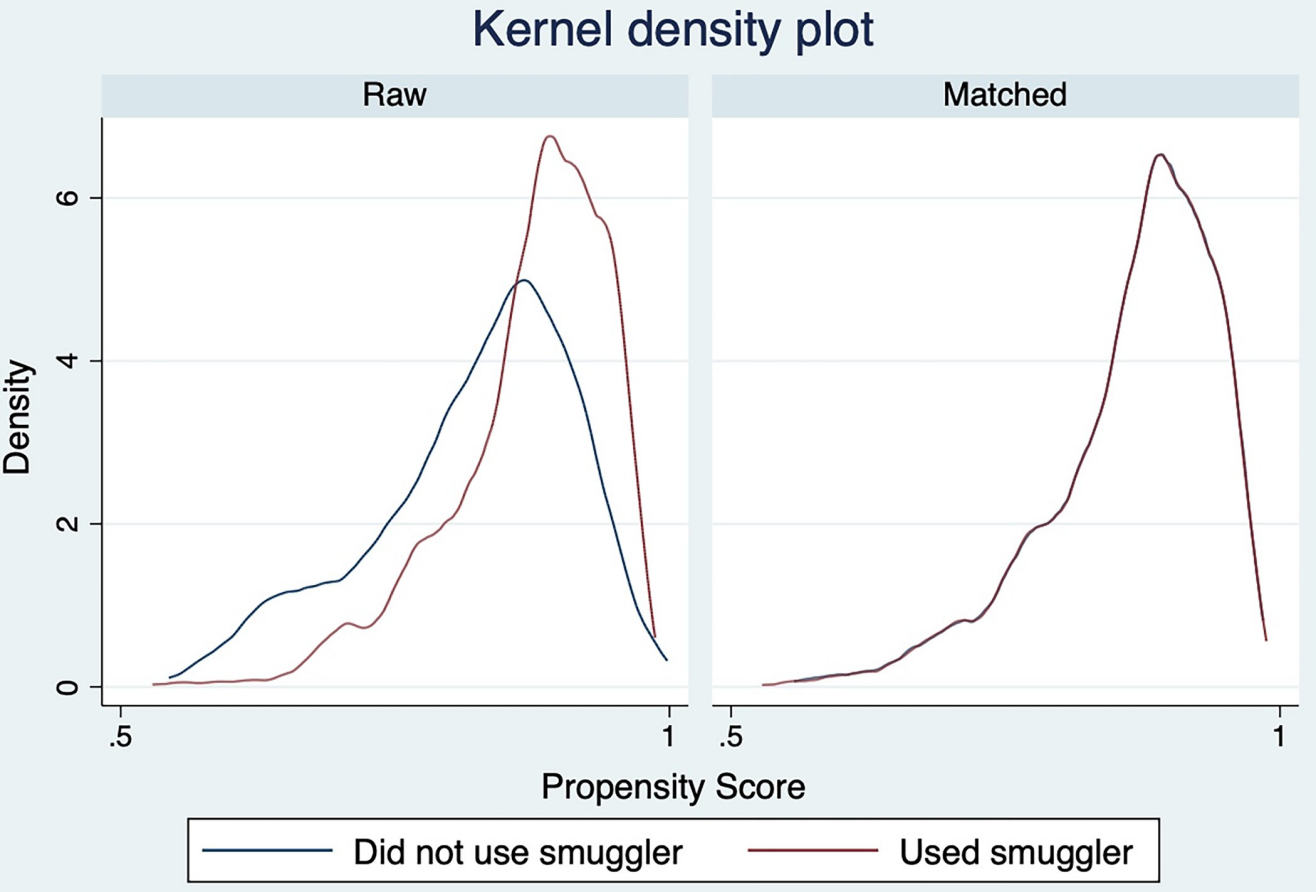
Kernel density plot by use of a smuggler to cross the US-Mexico border.

Our analysis strategy used multiple quasi-experimental methods to account for the imbalance of confounders across smuggler use. Entropy balancing weighting is a method that reweights the data to match the means, variance, and skewness of the covariates by the exposure variable [17]. Like propensity score matching, entropy balance weighting isolates the effect of undocumented immigrants who used smugglers to those who did not by creating a balance of covariates on the exposure variable. We included the MMP survey weight as a covariate for entropy balancing and then multiplied the entropy balancing weights by the MMP survey weights to produce population estimates from our logistic regression models [18]. Even though entropy balance weighting has been found to be more flexible and robust than propensity score matching, to provide a robustness check on our findings, we also estimated the average treatment effect from logit models using both propensity score matching and inverse-probability-weighted regression adjustment [19–21]. We included a balance box plot (Fig 1) and a kernel density plot (Fig 2) for the raw and balanced data to demonstrate the sample balance on the measured covariates. We used ebalance, a user-written program, to implement entropy balance weighting in STATA MP 17 [22]. A p-value less than 0.05 was defined as statistically significant using a 2-sided hypothesis test. The study followed Strengthening the Reporting of Observational Studies in Epidemiology (STROBE) guidelines for cross-sectional studies.

## Results

Table 1 provides the weighted characteristics of the study sample prior to creating covariate balance using entropy balance weighting and propensity score matching. Among undocumented immigrant adults, 87% used smugglers to cross the US-Mexico border. Most undocumented immigrants were less than 50 years old (66%), male (97%), married (80%), completed 6 years or less of education (59%), never smoked (62%), non-obese (78%), homeowners (74%), owned a vehicle (58%), did not own a business (60%), and migrated to the United States only once (61%). Among these variables, there were no significant differences in characteristics between immigrants that used a smuggler to cross the US-Mexico border other than age.

**Table 1:** Descriptive statistics of undocumented immigrant adults stratified by use of smugglers to cross the US-Mexico border: Mexican Migration Project 2007–19.

	Overall	Used Smuggler	Did Not Use Smuggler
N	1563	1353	210
%	100	87	13
Age, %			
18–34	20	21	15
35–49	46	47	38
50–64	25	24	30
65+	9	8	17
Sex, %			
Male	97	97	97
Female	3	3	3
Marital status, %			
Married	80	80	80
Not married	20	20	20
Education, %			
<6 years	26	25	31
6 years	33	34	33
7–11 years	29	30	24
12+ years	11	11	12
Ever smoked, %			
Yes	38	37	41
No	62	63	59
Obesity status, %			
Yes	22	21	25
No	78	79	75
Home ownership, %			
Yes	74	74	75
No	26	26	25
Vehicle ownership, %			
Yes	58	58	59
No	42	42	41
Business ownership, %			
Yes	40	41	36
No	60	59	64
US Migrations, %			
1	61	60	65
2+	39	40	35

Table 2 provides the average treatment effects estimated from multivariate logit regression using entropy balance weighting, propensity score matching, and inverse-probability-weighted regression adjustment. Undocumented immigrant adults that used a smuggler to cross the US-Mexico border reported a 4.7% higher prevalence of emotional or psychiatric problems compared to undocumented immigrant adults that did not use a smuggler (7.6% versus 2.9%). The results were nearly identical for each estimation method. All analyses were statistically significant as indicated by the 95% confidence intervals.

**Table 2:** Average treatment effect of emotional or psychiatric problems by use of smugglers to cross the US-Mexico border among undocumented immigrant adults: Mexican Migration Project, 2007–2019, n = 1,563.

Estimation Method	%	Confidence Interval
Entropy balance weighting	4.7	2.0–7.5
Propensity score matching	4.7	2.6–6.7
Inverse-probability-weighted regression adjustment	4.5	1.8–7.3

Note: Estimates for use of a smuggler to cross the US-Mexico border were derived from logit model and adjusted for age, sex, education, marital status, smoking, obesity, home ownership, vehicle ownership, business ownership, and number of US migrations.

## Discussion

Our quasi-experimental analysis of a nationally representative survey of Mexican citizens indicated that undocumented immigrants who used smugglers to cross the US-Mexico border may be at greater risk for emotional or psychiatric problems. Prior research had found that undocumented immigrants have worse health upon return to Mexico [10]. Specifically, there has been evidence of higher prevalence of mental health problems and diabetes [11, 12]. The increased prevalence is not likely due to a selection effect because healthier Mexicans were more likely to migrate to the United States and there has not been a difference found by documentation status [10, 11]. Therefore, we add to the evidence base by demonstrating a potential link by mode of border crossing, which is consistent with evidence that use of a smuggler may be a factor that is associated with heightened vulnerability to health risks [13, 14]. More research is needed to better understand the mechanisms linking the use of a smuggler and later health risks. Based on other studies of undocumented immigrants in the US, barriers to accessing healthcare and changes in health behaviors may be plausible mechanisms [5–9, 23–26].

The strength of the study is the use of a nationally representative survey of persons living in Mexico with detailed information on migration history that was analyzed using statistical matching procedures to isolate the effect of using a smuggler. However, our study using these data must be interpreted within the limitations of the data. Mental health was measured by a self-reported question asking respondents about the prevalence of emotional or psychiatric problems. Unfortunately, the MMP does not ask about mental health symptoms (e.g., Center for Epidemiological Studies Depression Scale) or precise indicators of mental health diagnoses (e.g., has a doctor ever diagnosed you with depression). By extension, the MMP does ask about year of diagnoses for health conditions, but most respondents report ‘don’t know’ which limits insight into the lag between migration and onset of mental health problems. Therefore, the results must be interpreted as a broad measure of prevalence of self-reported mental health problems, thereby reducing comparability with other mental health research. However, the data provide a unique insight into the association of human smuggling with the mental health status of undocumented immigrants to the US and calls for further research using standard instruments of mental health for this socially vulnerable community.

In conclusion, undocumented immigrants vary in their socioeconomic and health challenges, and our study focused on Mexican citizens suggests that modality of ingress into the United States should be considered a risk factor for mental health problems [10–12]. Understanding immigrants’ unique circumstances can provide insights into future investigations into equitable access to health care for vulnerable populations. Limitations in available data for undocumented immigrants to the US suggest the need for enhanced federal and state research support by increasing the funding for existing studies like the MMP and funding new studies targeting the public health and health care needs of a population that annually exceeds 10 million in the US [15, 27]. Moreover, US public policies on immigration may require greater attention given the long-term health consequences for Mexican citizens [15].
